# Supplementary material for: Advancing diagnostic performance and clinical usability of neural networks via adversarial training and dual batch normalization
Source: Nat Commun. 2021 Jul 14;12:4315. doi: 10.1038/s41467-021-24464-3 (PMC8280105; doi:10.1038/s41467-021-24464-3)
Supplement: Supplementary file 1 — Supplementray Information [file 41467_2021_24464_MOESM1_ESM.pdf]

# Supplementary Information

**Supplementary Table 1. Data characteristics of ChestX-ray8 and CheXpert dataset**

| | ChestX-ray8 dataset | CheXpert dataset |
|---|---|---|
| Number of patient radiographs | 112,120 | 224,316 |
| Number of patients | 30,805 | 65,240 |
| Age, mean (SD), years | 46.9 (16.6) | 60.7 (18.4) |
| Percentage of females (%) | 43.5% | 40.6% |
| Number of pathology labels | 8 | 14 |
| Cardiomegaly | 2,776 | 30,092 |
| Edema | 2,303 | 61,493 |
| Effusion | 13,317 | 86,477 |
| Pneumothorax | 5,302 | 20,401 |
| Atelectasis | 11,559 | 59,583 |
| Consolidation | 4,667 | 12,983 |
| Pneumonia | 1,431 | 20,656 |

The seven overlapping labels between the CheXpert and ChestX-ray8 datasets are listed in the table above. Note that pathological radiographs seem to be more common in CheXpert dataset due to the differences in the labeling process [1]. Abbreviations: SD, standard deviation.

**Supplementary Table 2. Individual and pooled ratings of radiologists in guiding them to the correct pathology on a scale from 0 (useless) to 5 (saliency map points clearly and unambiguously to the correct pathology)**

| | Dataset | Score for SSM | Score for SSBN | Score for SDBN | Friedman test |
|---|---|---|---|---|---|
| Radiologist 1 (8 years) | X-ray | $0.32 \pm 0.51$ | $1.56 \pm 0.84$ | $2.12 \pm 1.10$ | $p = 2.1 \cdot 10^{-34}$ |
| | MRI | $0.19 \pm 0.39$ | $0.39 \pm 0.57$ | $1.15 \pm 0.92$ | $p = 2.1 \cdot 10^{-21}$ |
| | CT | $0.65 \pm 0.50$ | $1.59 \pm 0.73$ | $1.73 \pm 0.83$ | $p = 1.5 \cdot 10^{-33}$ |
| Radiologist 2 (8 years) | X-ray | $1.53 \pm 1.16$ | $2.86 \pm 0.51$ | $3.30 \pm 0.83$ | $p = 1.1 \cdot 10^{-25}$ |
| | MRI | $1.65 \pm 0.61$ | $1.05 \pm 0.78$ | $2.70 \pm 1.22$ | $p = 3.2 \cdot 10^{-21}$ |
| | CT | $1.59 \pm 0.83$ | $2.70 \pm 1.08$ | $2.93 \pm 1.18$ | $p = 5.0 \cdot 10^{-23}$ |
| Radiologist 3 (8 years) | X-ray | $0.29 \pm 0.57$ | $2.47 \pm 1.64$ | $3.10 \pm 2.00$ | $p = 7.9 \cdot 10^{-30}$ |
| | MRI | $0.32 \pm 0.62$ | $0.54 \pm 1.21$ | $2.34 \pm 1.97$ | $p = 8.7 \cdot 10^{-20}$ |
| | CT | $0.11 \pm 0.31$ | $2.50 \pm 2.13$ | $2.67 \pm 2.14$ | $p = 1.6 \cdot 10^{-22}$ |
| Radiologist 4 (6 years) | X-ray | $1.03 \pm 1.21$ | $2.49 \pm 1.32$ | $2.52 \pm 1.34$ | $p = 4.8 \cdot 10^{-19}$ |
| | MRI | $0.24 \pm 0.51$ | $1.24 \pm 1.30$ | $2.83 \pm 1.46$ | $p = 2.0 \cdot 10^{-28}$ |
| | CT | $0.37 \pm 0.58$ | $2.92 \pm 1.83$ | $3.10 \pm 1.72$ | $p = 2.7 \cdot 10^{-30}$ |
| Radiologist 5 (5 years) | X-ray | $0.14 \pm 0.40$ | $1.90 \pm 1.42$ | $2.50 \pm 1.74$ | $p = 4.2 \cdot 10^{-29}$ |
| | MRI | $0.20 \pm 0.49$ | $0.40 \pm 1.09$ | $1.90 \pm 1.71$ | $p = 1.3 \cdot 10^{-20}$ |
| | CT | $0.06 \pm 0.24$ | $2.11 \pm 1.91$ | $2.29 \pm 1.91$ | $p = 2.5 \cdot 10^{-22}$ |
| Radiologist 6 (5 years) | X-ray | $0.13 \pm 0.34$ | $1.92 \pm 1.45$ | $2.57 \pm 1.76$ | $p = 3.3 \cdot 10^{-29}$ |
| | MRI | $0.09 \pm 0.29$ | $0.81 \pm 1.11$ | $2.11 \pm 1.31$ | $p = 2.6 \cdot 10^{-26}$ |
| | CT | $0.04 \pm 0.20$ | $2.08 \pm 1.85$ | $2.25 \pm 1.92$ | $p = 9.9 \cdot 10^{-23}$ |
| All radiologists | X-ray | $0.57 \pm 0.94$ | $2.20 \pm 1.33$ | $2.69 \pm 1.56$ | $p = 1.0 \cdot 10^{-160}$ |
| | MRI | $0.49 \pm 0.74$ | $0.74 \pm 1.09$ | $2.17 \pm 1.57$ | $p = 1.7 \cdot 10^{-118}$ |
| | CT | $0.47 \pm 0.73$ | $2.32 \pm 1.72$ | $2.50 \pm 1.74$ | $p = 7.0 \cdot 10^{-150}$ |

One-sided Friedman tests were used to the p-value.

**Supplementary Table 3. Comparison of standard and robust models on Luna16 test set**

| Prediction | ROC-AUC (95% CI) | p-value | Sensitivity (95% CI) | p-value | Specificity (95% CI) | p-value |
|---|---|---|---|---|---|---|
| **Tumor malignancy** | | | | | | |
| Standard model | 0.952 (0.946, 0.959) | - | 0.846 (0.827, 0.865) | - | 0.933 (0.923, 0.943) | - |
| Robust model | 0.877 (0.866, 0.888) | 0.008 | 0.779 (0.757, 0.800) | 0.240 | 0.810 (0.794, 0.826) | 0.065 |
| Robust model, with dual batch norms | 0.955 (0.949, 0.961) | 0.472 | 0.893 (0.878, 0.909) | 0.308 | 0.878 (0.864, 0.891) | 0.275 |

One-sided permutation tests were used to the p-value. Abbreviations: ROC-AUC, the area under the receiver operating characteristic curve; CI, confidence interval.

**Supplementary Table 4. Comparison of standard and robust models on kneeMRI test set**

| Prediction | ROC-AUC (95% CI) | p-value | Sensitivity (95% CI) | p-value | Specificity (95% CI) | p-value |
|---|---|---|---|---|---|---|
| **Healthy ACL** | | | | | | |
| Standard model | 0.824 (0.807, 0.841) | - | 0.788 (0.773, 0.803) | - | 0.730 (0.701, 0.758) | - |
| Robust model | 0.642 (0.622, 0.662) | <0.001 | 0.697 (0.680, 0.714) | 0.256 | 0.540 (0.508, 0.573) | 0.056 |
| Robust model, with dual batch norms | 0.825 (0.808, 0.841) | 0.487 | 0.788 (0.773, 0.803) | 0.493 | 0.717 (0.688, 0.745) | 0.467 |
| **Partially injured ACL** | | | | | | |
| Standard model | 0.742 (0.721, 0.763) | - | 0.660 (0.625, 0.696) | - | 0.735 (0.719, 0.750) | - |
| Robust model | 0.634 (0.614, 0.655) | 0.039 | 0.536 (0.499, 0.573) | 0.213 | 0.675 (0.658, 0.691) | 0.307 |
| Robust model, with dual batch norms | 0.741 (0.720, 0.761) | 0.482 | 0.626 (0.590, 0.661) | 0.393 | 0.731 (0.716, 0.747) | 0.485 |
| **Completely ruptured ACL** | | | | | | |
| Standard model | 0.921 (0.909, 0.933) | - | 0.945 (0.915, 0.975) | - | 0.802 (0.789, 0.815) | - |
| Robust model | 0.601 (0.557, 0.644) | <0.001 | 0.610 (0.573, 0.674) | 0.031 | 0.645 (0.629, 0.660) | 0.218 |
| Robust model, with dual batch norms | 0.918 (0.908, 0.929) | 0.499 | 1.000 (1.000) | 0.434 | 0.798 (0.785, 0.811) | 0.494 |

One-sided permutation tests were used to the p-value. Abbreviations: ACL, anterior cruciate ligament; ROC-AUC, the area under the receiver operating characteristic curve; CI, confidence interval.

**Supplementary Table 5. Comparison of standard and robust models on CheXpert test set**

| Prediction | ROC-AUC (95% CI) | p-value | Sensitivity (95% CI) | p-value | Specificity (95% CI) | p-value |
|---|---|---|---|---|---|---|
| **Cardiomegaly** | | | | | | |
| Standard model | 0.798 (0.782, 0.814) | - | 0.727 (0.703, 0.752) | - | 0.757 (0.741, 0.774) | - |
| Robust model | 0.826 (0.812, 0.839) | 0.348 | 0.773 (0.750, 0.796) | 0.342 | 0.728 (0.711, 0.745) | 0.420 |
| Robust model, with dual batch norms | 0.853 (0.841, 0.866) | 0.177 | 0.743 (0.718, 0.767) | 0.426 | 0.809 (0.794, 0.824) | 0.334 |
| **Edema** | | | | | | |
| Standard model | 0.937 (0.929, 0.946) | - | 0.881 (0.859, 0.904) | - | 0.881 (0.870, 0.893) | - |
| Robust model | 0.880 (0.868, 0.893) | 0.198 | 0.881 (0.858, 0.904) | 0.500 | 0.744 (0.728, 0.759) | 0.161 |
| Robust model, with dual batch norms | 0.927 (0.918, 0.936) | 0.457 | 0.928 (0.911, 0.946) | 0.401 | 0.831 (0.818, 0.845) | 0.361 |
| **Consolidation** | | | | | | |
| Standard model | 0.918 (0.909, 0.927) | - | 0.937 (0.918, 0.957) | - | 0.806 (0.792, 0.820) | - |
| Robust model | 0.928 (0.910, 0.936) | 0.471 | 0.813 (0.781, 0.844) | 0.225 | 0.865 (0.853, 0.876) | 0.355 |
| Robust model, with dual batch norms | 0.936 (0.928, 0.943) | 0.396 | 0.906 (0.883, 0.929) | 0.465 | 0.841 (0.828, 0.854) | 0.422 |
| **Pneumonia** | | | | | | |
| Standard model | 0.857 (0.837, 0.876) | - | 1.000 (1.000) | - | 0.727 (0.713, 0.741) | - |
| Robust model | 0.810 (0.785, 0.834) | 0.373 | 0.750 (0.681, 0.819) | 0.203 | 0.737 (0.723, 0.751) | 0.498 |
| Robust model, with dual batch norms | 0.794 (0.768, 0.820) | 0.350 | 1.000 (1.000) | - | 0.588 (0.572, 0.604) | 0.304 |
| **Atelectasis** | | | | | | |
| Standard model | 0.808 (0.794, 0.822) | - | 0.787 (0.765, 0.808) | - | 0.756 (0.739, 0.773) | - |
| Robust model | 0.751 (0.736, 0.766) | 0.161 | 0.760 (0.737, 0.782) | 0.370 | 0.661 (0.643, 0.680) | 0.247 |
| Robust model, with dual batch norms | 0.818 (0.804, 0.832) | 0.426 | 0.800 (0.779, 0.821) | 0.455 | 0.772 (0.755, 0.789) | 0.454 |
| **Pneumothorax** | | | | | | |
| Standard model | 0.865 (0.836, 0.895) | - | 0.714 (0.636, 0.792) | - | 0.795 (0.782, 0.808) | - |
| Robust model | 0.586 (0.537, 0.635) | 0.039 | 0.572 (0.487, 0.657) | 0.369 | 0.631 (0.615, 0.646) | 0.246 |
| Robust model, with dual batch norms | 0.791 (0.748, 0.834) | 0.330 | 0.569 (0.484, 0.655) | 0.178 | 0.995 (0.993, 0.997) | 0.179 |
| **Effusion** | | | | | | |
| Standard model | 0.912 (0.903, 0.921) | - | 0.781 (0.758, 0.805) | - | 0.870 (0.857, 0.882) | - |
| Robust model | 0.919 (0.910, 0.927) | 0.449 | 0.828 (0.807, 0.849) | 0.409 | 0.870 (0.857, 0.882) | 0.500 |
| Robust model, with dual batch norms | 0.931 (0.923, 0.939) | 0.383 | 0.797 (0.774, 0.820) | 0.429 | 0.891 (0.879, 0.903) | 0.423 |

One-sided permutation tests were used to the p-value. Abbreviations: ROC-AUC, the area under the receiver operating characteristic curve; CI, confidence interval.

**Supplementary Table 6. Lipschitzness of model loss on CheXpert test set**

| model name | Lipschitz constant of loss |
|---|---|
| Standard | $0.706 \pm 0.950$ |
| Advers. | $0.360 \pm 0.506$ |

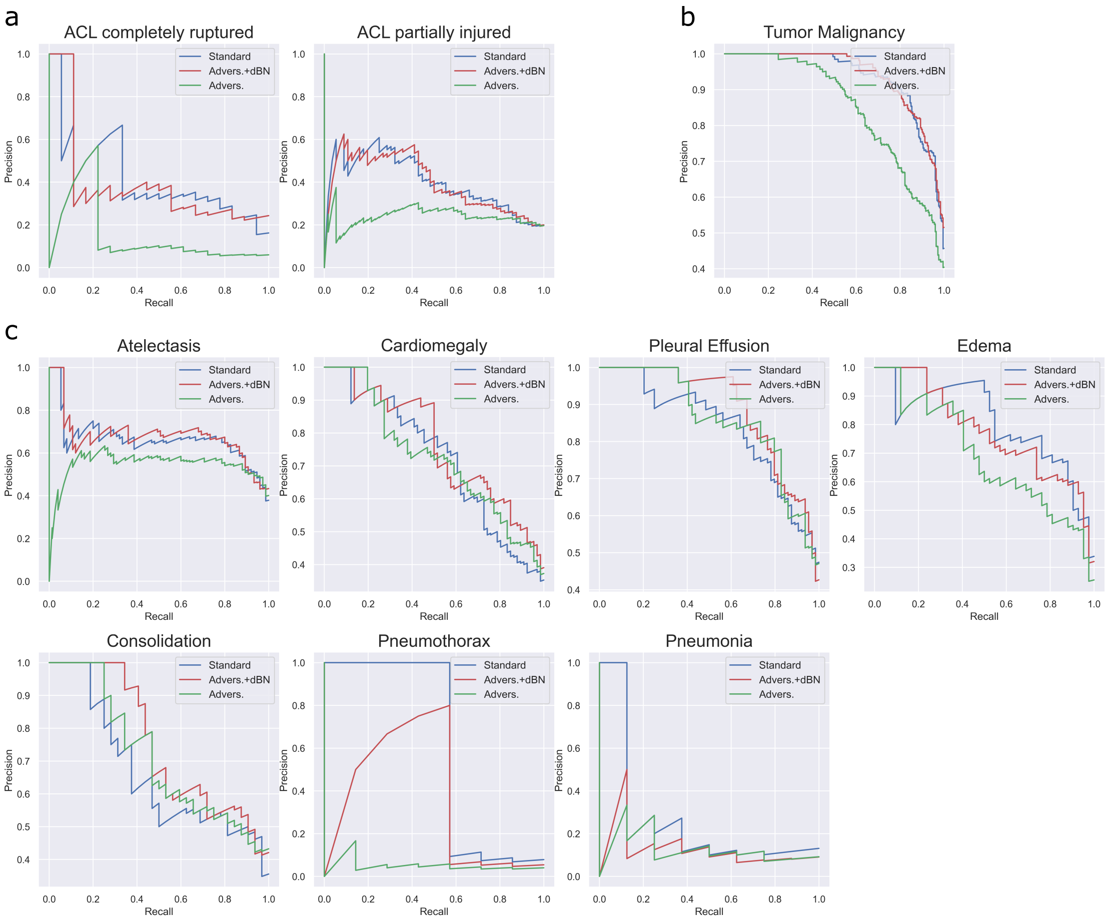


**Supplementary Fig. 1. Precision-recall analysis also suggests that the setting of dual batch norms is beneficial to improve the performance of robust models.** We compared the classification performance of three models, namely, standard, adversarially trained, and adversarially trained with dual batch norms, against the test sets of the ground truth in the precision-recall space. The test sets were collected from Rijeka MRI dataset (a), Luna16 CT dataset (b), and CheXpert X-rays dataset (c). Note that the adversarially trained model performs comparable to the standard model only when employing dual batch norms.

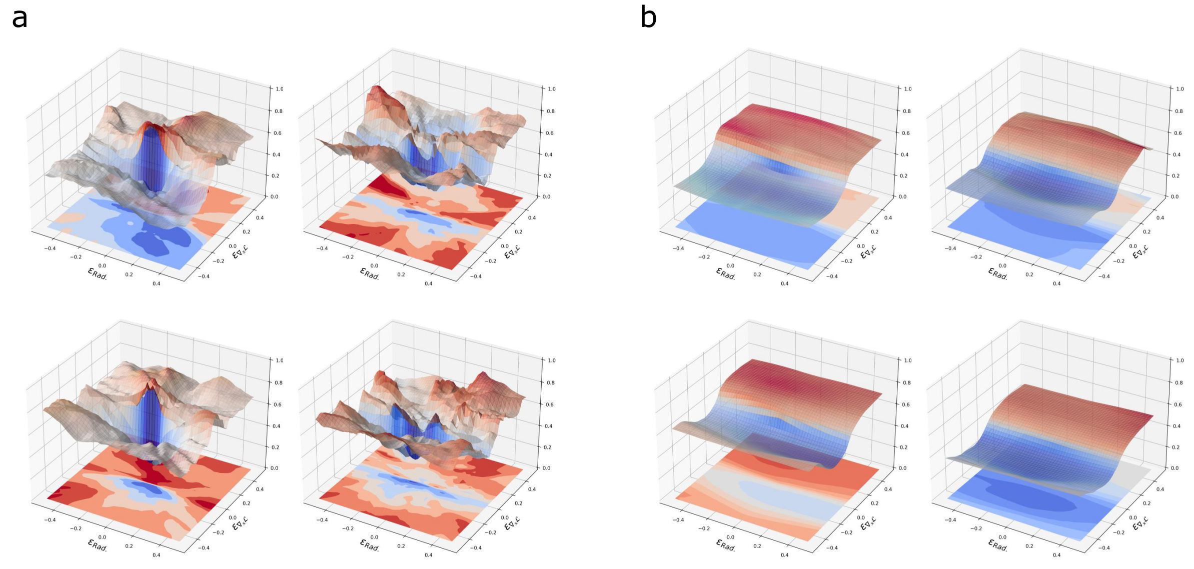


**Supplementary Fig. 2. Adversarial training regularizes the model via minimizing its Jacobian and Hessian matrices.** The loss surface of a model that is only trained on unaltered images (no adversarial augmentation, i.e. model trained in a standard manner) (a) and a model trained solely with adversarial images with $\varepsilon$=0.005 (b) for the CheXpert dataset on four test images. We display the cross-entropy loss projected on one random $\varepsilon_{Rad}$ and one gradient $\varepsilon_{\nabla_x L}$ direction in the input space. Due to regularization, the robust model b has a loss that is smooth both in the gradient direction and in the random direction, whereas the loss surface of the standard model a changes rapidly both in all directions.

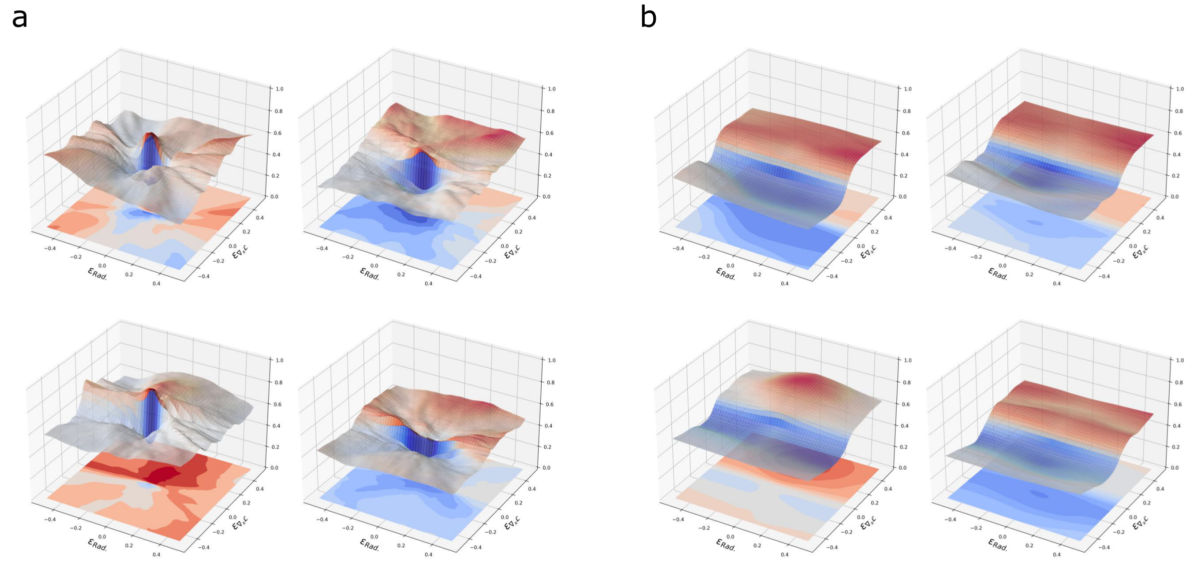


**Supplementary Fig. 3. Loss landscape relates closely to batch norm layers.** The loss surface of an adversarially augmented model ($\varepsilon$=0.005) with $BN_{std}$ (a) and $BN_{adv}$ (a) for CheXpert on four test images. As a result of different reparameterization ($\gamma$ or $\gamma'$), the loss surfaces are different in (a) and (b).

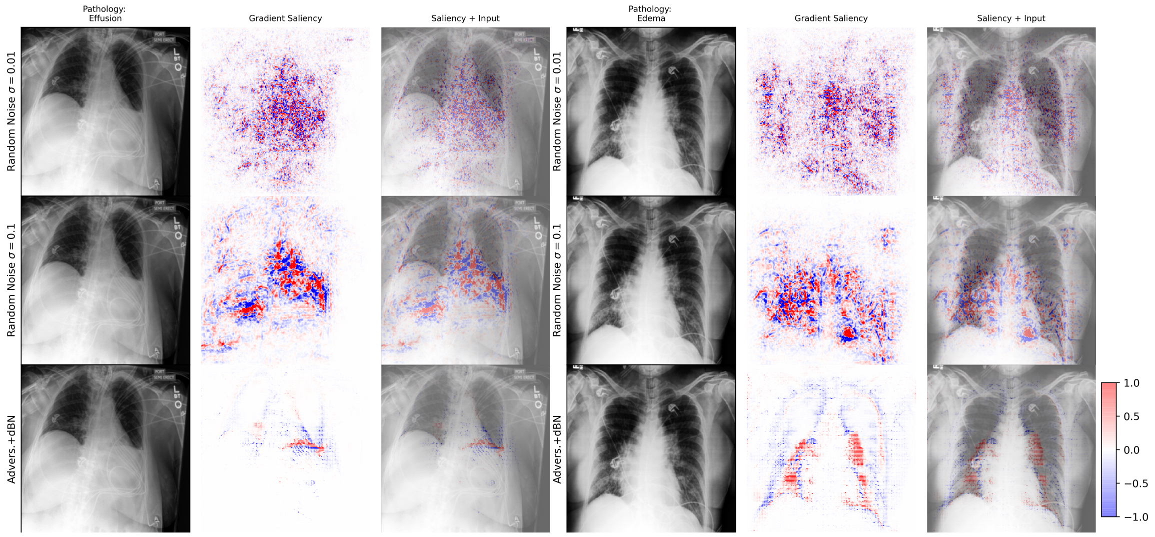


**Supplementary Fig. 4. The loss gradient with respect to input pixels of random and adversarial noise augmented models.** We additionally trained three models with medium variance Gaussian noise ($\sigma$=0.01), large variance Gaussian noise ($\sigma$=0.1), and adversarial perturbations ($\varepsilon$=0.005). As expected, gradients of models augmented with added random noise are noisy and semantically meaningless, whereas, the adversarially trained model yields interpretable gradient saliencies. Both the area of effusion (left image column, left patient side near diaphragm - note that the left patient side corresponds to the right image side) and edema (right image column, central portion of the image) are accurately depicted by the adversarial training with dual batch norm contrary to the other models.

## GradCAM Visualization

We also investigated the effect of adversarial training on GradCAM, see Fig. 5. GradCAM is a technique with limited resolution: it points to the approximate image region, but fails to exactly delineate smaller image regions responsible for the diagnosis. Hence, the difference between adversarially trained networks with dual batch norms and standard networks was less pronounced and the diagnostic rating was lower in general: Based on the rating standard in table 2 of the manuscript, we assessed the diagnostic relevance of 100 randomly selected chest X-rays and their gradient saliencies by one radiologist. The evaluation scores were 0.83±0.67 and 0.98±0.72 for the GradCAM with standard training and the GradCAM with dual batch norm adversarial training respectively.

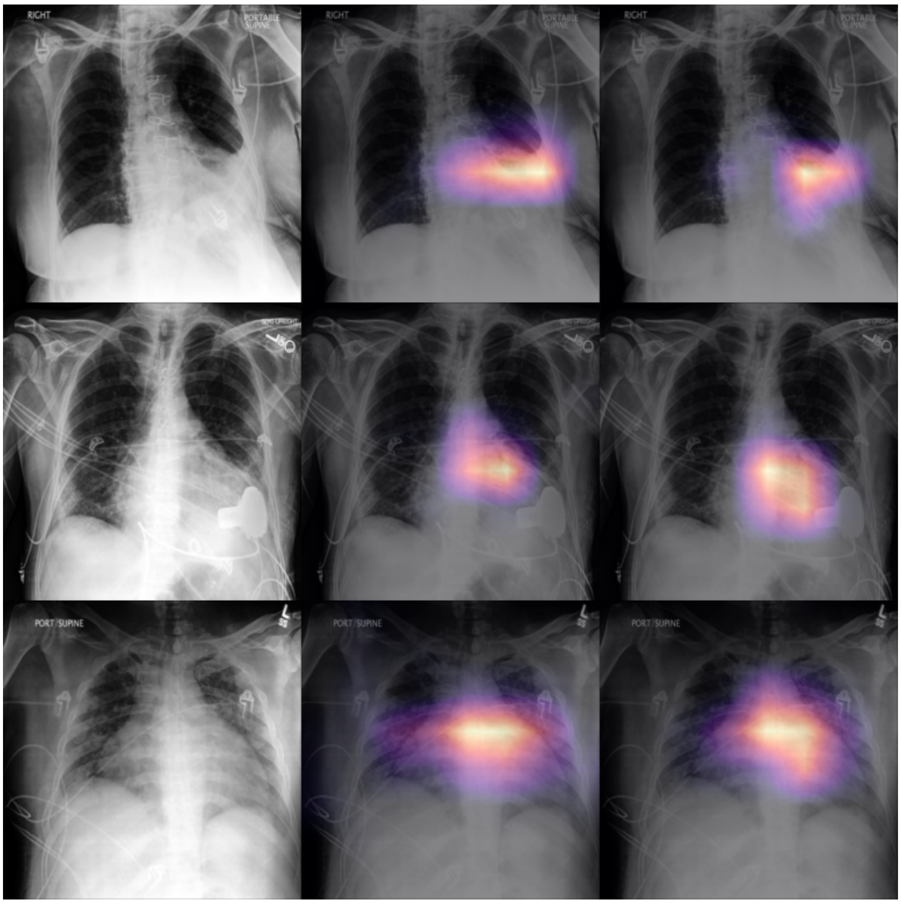

**Supplementary Fig. 5. The robust model locate abnormalities in radiographs using GradCAM.** Both standard (2nd column) and adversarially trained (3rd column) models are able to localize abnormalities in chest radiographs. Patients from CheXpert test set with pleural effusion, cardiomegaly, and edema are listed in 1-3 rows, respectively. GradCAMs are generated by the most confident correct class of the model.

## Robustness of Adversarially Augmented Model

We investigated the robustness of the adversarially augmented models $BN_{std}$ and $BN_{adv}$ (training $\varepsilon$=0.005) for CheXpert, Rijeka, and Luna datasets. The trained parameters of $BN_{std}$ ($\gamma$) are always kept secret while parameters of $BN_{adv}$ ($\gamma'$) and remaining parameters ($\theta'$) are available to the public. Here, we applied black and white box attacks to $BN_{std}$ and $BN_{adv}$ separately as PGD adversaries are exclusively generated via robust batch norms, e.g., $\gamma'$ and $\theta'$. Indeed we found, that the performance of $BN_{std}$ remains acceptable when facing transferred PGD attacks with reasonable amplitudes ($\varepsilon$ up to 0.01), see Fig. 6.

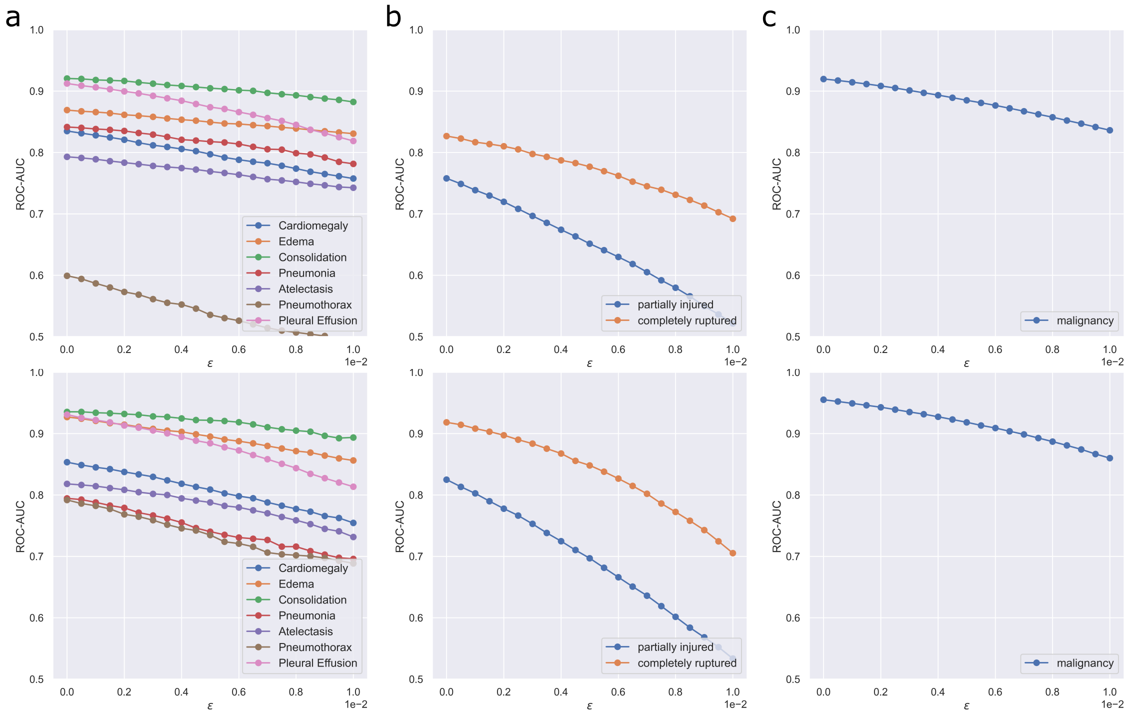


**Supplementary Fig. 6. Robustness of adversarially augmented model.** The classification performance of a adversarially augmented model ($\varepsilon$=0.005) with $\mathrm{BN}_{\mathrm{adv}}$ (1st row) and $\mathrm{BN}_{\mathrm{std}}$ (2nd row) on CheXpert (a), Rijeka (b), and LUNA (c) test sets. The trained $\mathrm{BN}_{\mathrm{std}}$ branch remains robust while facing transferred attacks from $\mathrm{BN}_{\mathrm{adv}}$.

## Linear CKA and Relation to Prior Work

The high correlation between features learned by robust layers is reflected by the overall high CKA value in Fig. 7 b and d of the manuscript. The linear CKA used in our study is defined in equation 8: Considering eigen-decomposition, we let $\lambda_X^i$ and $\mathbf{u}_X^i$ be the i$^{\text{th}}$ eigenvalue and eigenvector of the dot products between the representations of the samples $XX^T$. The linear CKA can be rewritten as [2]:

$$\mathrm{CKA}(X,Y) = \frac{\sum_i \sum_j \lambda_X^i \lambda_Y^j}{\sqrt{\sum_i (\lambda_X^i)^2}\sqrt{\sum_j (\lambda_Y^j)^2}} \left\langle \mathbf{u}_X^i, \mathbf{u}_Y^i \right\rangle^2 . \tag{1}$$

The term $\left\langle \mathbf{u}_X^i, \mathbf{u}_Y^i \right\rangle$ reveals that the overall high CKAs of a robust model is a result of feature eigenvectors of adversaries trained layers that tend to align to a similar direction. Via the lens of dual batch norms (Fig. 7 c and d), the link between batch norms and the model accuracy is reflected by the inner product change of feature eigenvectors when switching between batch norm branches, i.e., $\mathrm{BN}_{\mathrm{std}}$ and $\mathrm{BN}_{\mathrm{adv}}$.

To find the link between feature similarity and robust learning, a previous work by Zhang, H, et al. [3] improved model robustness against $l_\infty$ attacks via optimizing similarities between features in the minibatch. Using the proposed feature-based adversarial training, they obtain a more regularized loss landscape and avoided label information leakage. However, to the best of our knowledge, no previous study compared the interlayer feature dependencies between standard and robust models. The importance of batch norms in adversarial vulnerability was studied in [4]. Instead of using simple models [4], our ResNet-50 model with dual batch norms offers the opportunity to inspect the influence of batch norms on robustness (loss landscape in Fig. 3) and accuracy (feature similarity in Fig. 7).

## Reparameterization for adversarial augmented training

Despite the domain is distinguishable among standard ($x$) and adversarial batches ($x^*$) [5], in our study, we also confirm different reparameterization is applied to those batches. By definition, Adversarial batches are

difficult to optimize when compared to their standard counterparts. To investigate, we constructed a simple fully-connected network with one hidden layer followed by two parallel batch norm layers (one for $x$ and one for $x^*$). We perform our analysis on Luna16 and MNIST [6] datasets in combination with different attack strength ($\varepsilon$). Recall, the Lipschitz constant of the loss, i.e., gradient magnitude $\|\hat{g}\|$ of a network with batch norms, is governed by reparameterization $\gamma$ [7]. Therefore, in adversarial batches, a smaller $\gamma$ is favorable as it corresponds to a smaller Lipschitzness of the loss function and thus facilitates optimization. As shown in Fig. 7 a-c and d-f, the learned $\gamma$ of adversarial batch norms shift to smaller magnitudes when applying stronger attacks, whereas the distribution of standard batch norm remains unchanged.

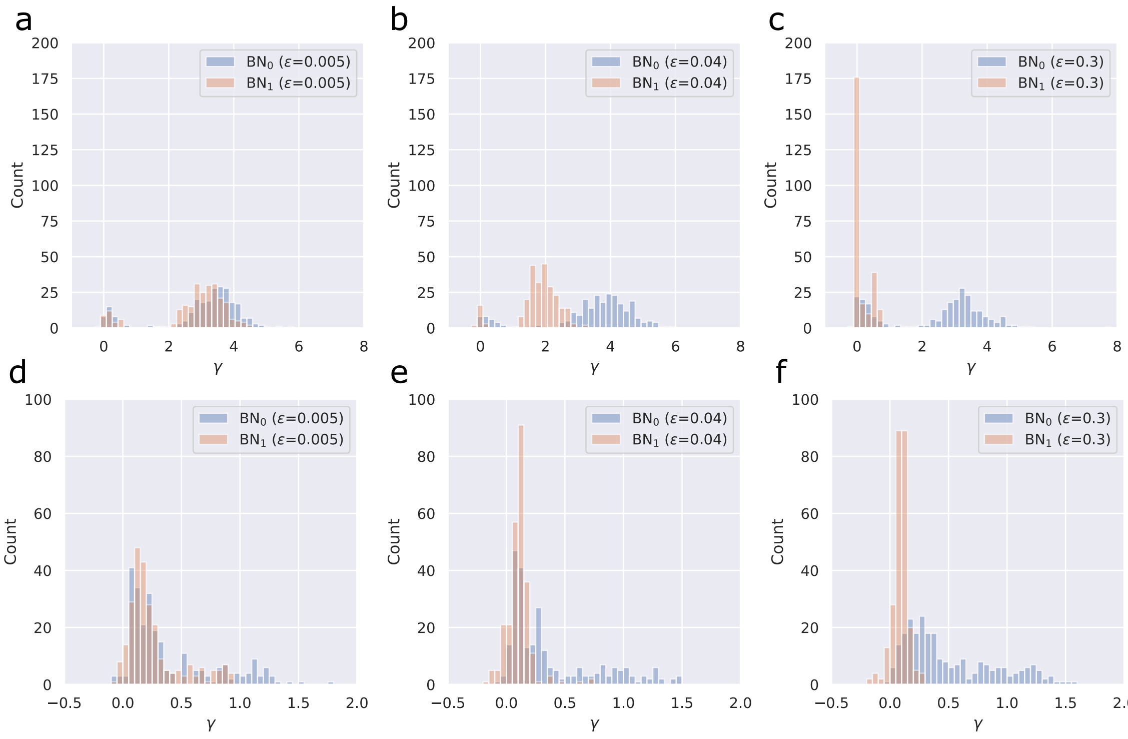


**Supplementary Fig. 7. A smaller magnitude of $\gamma$ was learned when increasing adversarial attack strength during training.** Analysis of the reparameterization of batch norms versus adversarial strength $\varepsilon = 0.005$, 0.04, and 0.3. A shallow network with just one hidden layer (n=256 neurons and ReLU activation) was trained on both MNIST (a-c) and Luna16 (d-f) datasets. We observe the distribution of adversarial batch $\gamma$ shifts towards zero under stronger adversarial attacks ($\varepsilon = 0.3$).

## The influence of perturbation strength on adversarial training

We also investigate how the performance of robust models changes when increasing adversarial attack strength. Under the setting of training via separate batch norm layers, the model performance is stable as no significant performance decrease was observed when increasing $\varepsilon$ from 0.005 to 0.04 (Fig. 8).

However, as visualized in Fig. 9, we observe the general learned Gabor filters (Fig. 9 b) are replaced by checkerboard-like filters (Fig. 9 c and d) when applying stronger adversarial attacks during training.

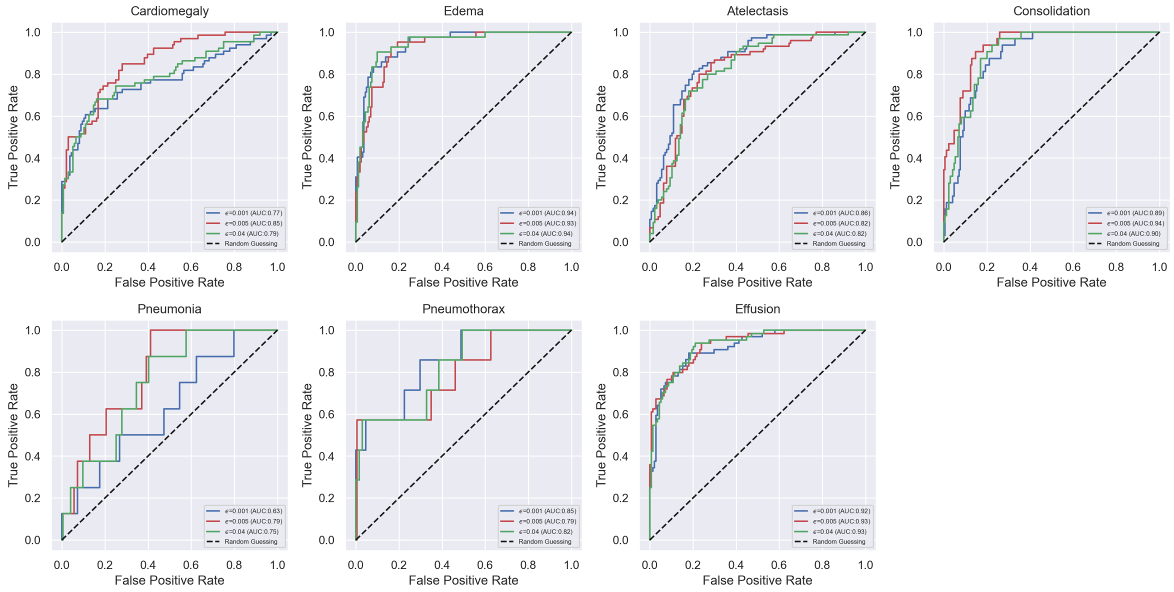


**Supplementary Fig. 8. Model performance versus training adversarial strength.** We observe that when training with different attack strength, i.e., $\varepsilon = 0.001$, 0.005, and 0.04, the performance of adversarial training with separate batch norms shows little difference.

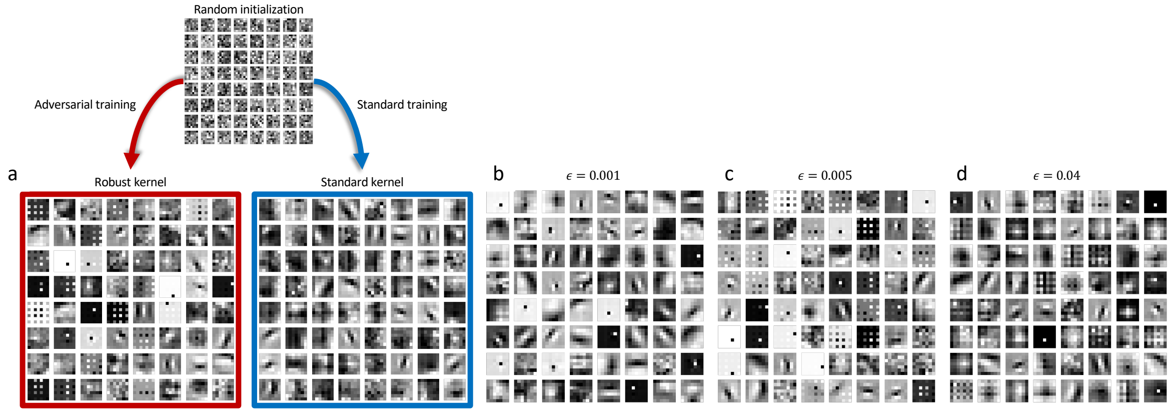


**Supplementary Fig. 9. The influence of training adversarial strength on learned convolutional kernels.** Visualization of Conv1 filters (7×7) shows Gabor edge detectors are typically learned through standard training a. In addition, by adversarial training, robust models learned Dirac-delta functions (kernels) to gain higher robustness [8] b. From b to d, we find that checkerboard patterns appear within trained kernels when increasing adversarial strength.

# Supplementary References


[1] Irvin, J. *et al.* Chexpert: A large chest radiograph dataset with uncertainty labels and expert comparison. In *Proceedings of the AAAI Conference on Artificial Intelligence*, vol. 33, 590–597 (2019).

[2] Kornblith, S., Norouzi, M., Lee, H. & Hinton, G. Similarity of neural network representations revisited. *arXiv preprint arXiv:1905.00414* (2019).

[3] Zhang, H. & Wang, J. Defense against adversarial attacks using feature scattering-based adversarial training. *arXiv preprint arXiv:1907.10764* (2019).

[4] Galloway, A., Golubeva, A., Tanay, T., Moussa, M. & Taylor, G. W. Batch normalization is a cause of adversarial vulnerability. *arXiv preprint arXiv:1905.02161* (2019).

[5] Xie, C. & Yuille, A. Intriguing properties of adversarial training at scale. *arXiv preprint arXiv:1906.03787* (2019).

[6] LeCun, Y. The mnist database of handwritten digits. *http://yann. lecun. com/exdb/mnist/* (1998).

[7] Santurkar, S., Tsipras, D., Ilyas, A. & Madry, A. How does batch normalization help optimization? In *Advances in Neural Information Processing Systems*, 2483–2493 (2018).

[8] Pérez, J. C. *et al.* Robust gabor networks. *arXiv preprint arXiv:1912.05661* (2019).
