# Supplementary material for: Advancing diagnostic performance and clinical usability of neural networks via adversarial training and dual batch normalization
Source: Nat Commun. 2021 Jul 14;12:4315. doi: 10.1038/s41467-021-24464-3 (PMC8280105; doi:10.1038/s41467-021-24464-3)
Supplement: Supplementary file 2 — Reporting Summary [file 41467_2021_24464_MOESM2_ESM.pdf]

nature research

Corresponding author(s): Volkmar Schulz

Last updated by author(s): Jun 11, 2021

# Reporting Summary

Nature Research wishes to improve the reproducibility of the work that we publish. This form provides structure for consistency and transparency in reporting. For further information on Nature Research policies, see our Editorial Policies and the Editorial Policy Checklist.

## Statistics

For all statistical analyses, confirm that the following items are present in the figure legend, table legend, main text, or Methods section.

| n/a | Confirmed | |
|---|---|---|
| ☐ | ☒ | The exact sample size (*n*) for each experimental group/condition, given as a discrete number and unit of measurement |
| ☐ | ☒ | A statement on whether measurements were taken from distinct samples or whether the same sample was measured repeatedly |
| ☐ | ☒ | The statistical test(s) used AND whether they are one- or two-sided<br>*Only common tests should be described solely by name; describe more complex techniques in the Methods section.* |
| ☒ | ☐ | A description of all covariates tested |
| ☐ | ☒ | A description of any assumptions or corrections, such as tests of normality and adjustment for multiple comparisons |
| ☐ | ☒ | A full description of the statistical parameters including central tendency (e.g. means) or other basic estimates (e.g. regression coefficient) AND variation (e.g. standard deviation) or associated estimates of uncertainty (e.g. confidence intervals) |
| ☐ | ☒ | For null hypothesis testing, the test statistic (e.g. *F*, *t*, *r*) with confidence intervals, effect sizes, degrees of freedom and *P* value noted<br>*Give P values as exact values whenever suitable.* |
| ☒ | ☐ | For Bayesian analysis, information on the choice of priors and Markov chain Monte Carlo settings |
| ☒ | ☐ | For hierarchical and complex designs, identification of the appropriate level for tests and full reporting of outcomes |
| ☒ | ☐ | Estimates of effect sizes (e.g. Cohen's *d*, Pearson's *r*), indicating how they were calculated |

*Our web collection on statistics for biologists contains articles on many of the points above.*

## Software and code

Policy information about availability of computer code

| | |
|---|---|
| Data collection | No software was used. |
| Data analysis | We use python python 3.6.5 (https://www.python.org), Numpy 1.16.0 (https://numpy.org/), Scipy 1.21.0 (https://www.scipy.org/), and pytorch 1.1 (https://pytorch.org/) to analysis the data. The analysis code can be found at https://github.com/peterhan91/Medical-Robust-Training. |

For manuscripts utilizing custom algorithms or software that are central to the research but not yet described in published literature, software must be made available to editors and reviewers. We strongly encourage code deposition in a community repository (e.g. GitHub). See the Nature Research guidelines for submitting code & software for further information.

## Data

Policy information about availability of data

All manuscripts must include a data availability statement. This statement should provide the following information, where applicable:

- Accession codes, unique identifiers, or web links for publicly available datasets
- A list of figures that have associated raw data
- A description of any restrictions on data availability

The two X-ray datasets used in this study are available in NIH ChestX-ray8 database and Stanford CheXpert database under accession code https://nihcc.app.box.com/v/ChestXray-NIHCC and https://stanfordmlgroup.github.io/competitions/chexpert. The MRI and CT datasets are available in Rijeka knee MRI database and LUNA16 database under accession code http://www.riteh.uniri.hr/~istajduh/projects/kneeMRI/ and https://luna16.grand-challenge.org/Data/. In addition, we use publicly available MNIST dataset http://yann.lecun.com/exdb/mnist/ for experiments in the supplementary section. All data needed to evaluate the findings in the paper are present in the paper and/or the supplementary material. Additional data related to this paper such as the detailed reader test data maybe requested from the authors.



April 2020

# Field-specific reporting

Please select the one below that is the best fit for your research. If you are not sure, read the appropriate sections before making your selection.

- [x] Life sciences
- [ ] Behavioural & social sciences
- [ ] Ecological, evolutionary & environmental sciences

For a reference copy of the document with all sections, see nature.com/documents/nr-reporting-summary-flat.pdf

# Life sciences study design

All studies must disclose on these points even when the disclosure is negative.

**Sample size**

A total number of four medical imaging datasets are used in this study: the CheXpert dataset, which has been released by Irvin et al. in January 2019 and contains 224,316 chest radiographs of 65,240 patients. Only 191,027 frontal radiographs are downloadable for model training. No sample size calculation was performed. We used the official data stratification provided by the data host. For testing, we compare the performance of the trained models on the official validation set of 202 scans on which the concurrence of diagnosis from three radiologists serves as ground truth.
Another X-ray dataset used in this study is the ChestX-ray8 dataset released by the National Institutes of Health (NIH) in 2017, containing 112,120 frontal radiographs of 30,805 unique patients. All available X-rays are used to develop and test the model. We randomly select 20% out of 112,120 radiographs, i.e., 6,187 patients and 22.433 radiographs, to form an external test set.
The MRI dataset used in this study is the kneeMRI dataset, which has been released by Stajduhar et al. in 2017 and contains 917 sagittal proton-density weighted knee scans from Clinical Hospital Centre Rijeka, Croatia. Three degrees of anterior cruciate ligament (ACL) injuries were recorded by radiologists in the Rijeka dataset: namely non-injured (692 scans), partially injured (172 scans), and completely ruptured (55 scans). According to ROI labels, we extracted a total of 3,081 diagnostic relevant MRI slices and randomly split them into 80% training, 10% development, and 10% testing.
Lastly, we investigated the applicability of adversarial training on CT data with the Luna16 dataset consisting of 888 CT scans with lung cancer ROI annotations. In total, a number of 6,691 lung cancer patches were extracted and randomly split into 80% training, 10% development, and 10% testing.
CheXpert and ChestX-ray 8 are considered as large 2D medical dataset up to date (Irvin et al., 2019; Wang et al., 2017).
For the case of volumetric dataset, such as CT and MRI datasets, Rijieka MRI and LUNA16 are the largest datasets with precise ROI labels.

**Data exclusions**

No data was excluded.

**Replication**

For the image analysis and statistical analysis code, we carefully checked the code and ran it several times (n>3) to verify the reproducibility of the code. We validated the experiments on external datasets. All code and data used in this study are made openly available for reproducibility.

**Randomization**

We separate the train and test dataset randomly. A separate group of patients were randomly selected before creation of the training and validation datasets as an independent test set which was kept separate during model development.

**Blinding**

The raters were blinded for test image generation (Fig. 4). It was done randomly by the python program.

# Reporting for specific materials, systems and methods

We require information from authors about some types of materials, experimental systems and methods used in many studies. Here, indicate whether each material, system or method listed is relevant to your study. If you are not sure if a list item applies to your research, read the appropriate section before selecting a response.

## Materials & experimental systems

| n/a | Involved in the study |
|---|---|
| x | Antibodies |
| x | Eukaryotic cell lines |
| x | Palaeontology and archaeology |
| x | Animals and other organisms |
| x | Human research participants |
| x | Clinical data |
| x | Dual use research of concern |

## Methods

| n/a | Involved in the study |
|---|---|
| x | ChIP-seq |
| x | Flow cytometry |
| x | MRI-based neuroimaging |
